# Analysis of arterial intimal hyperplasia: review and hypothesis

**DOI:** 10.1186/1742-4682-4-41

**Published:** 2007-10-31

**Authors:** Vladimir M Subbotin

**Affiliations:** 1 Mirus Bio Corporation, 505 S Rosa Rd, Madison, Wisconsin, 53719, USA

## Abstract

**Background:**

Despite a prodigious investment of funds, we cannot treat or prevent arteriosclerosis and restenosis, particularly its major pathology, arterial intimal hyperplasia. A cornerstone question lies behind all approaches to the disease: what causes the pathology?

**Hypothesis:**

I argue that the question itself is misplaced because it implies that intimal hyperplasia is a novel pathological phenomenon caused by new mechanisms. A simple inquiry into arterial morphology shows the opposite is true. The normal multi-layer cellular organization of the *tunica intima *is identical to that of diseased hyperplasia; it is the standard arterial system design in all placentals at least as large as rabbits, including humans. Formed initially as one-layer endothelium lining, this phenotype can either be maintained or differentiate into a normal multi-layer cellular lining, so striking in its resemblance to diseased hyperplasia that we have to name it "benign intimal hyperplasia". However, normal or "benign" intimal hyperplasia, although microscopically identical to pathology, is a controllable phenotype that rarely compromises blood supply. It is remarkable that each human heart has coronary arteries in which a single-layer endothelium differentiates early in life to form a multi-layer intimal hyperplasia and then continues to self-renew in a controlled manner throughout life, relatively rarely compromising the blood supply to the heart, causing complications requiring intervention only in a small fraction of the population, while all humans are carriers of benign hyperplasia. Unfortunately, this fundamental fact has not been widely appreciated in arteriosclerosis research and medical education, which continue to operate on the assumption that the normal arterial intima is always an "ideal" single-layer endothelium. As a result, the disease is perceived and studied as a new pathological event caused by new mechanisms. The discovery that normal coronary arteries are morphologically indistinguishable from deadly coronary arteriosclerosis continues to elicit surprise.

**Conclusion:**

Two questions should inform the priorities of our research: (1) what controls switch the single cell-layer intimal phenotype into normal hyperplasia? (2) how is normal (benign) hyperplasia maintained? We would be hard-pressed to gain practical insights without scrutinizing our premises.

## Background

Most publications on coronary artery disease discuss progress achieved. However, there is an alternative perception of the problem, rarely enunciated in established medical journals: the stunning failure of contemporary medicine to treat cardiovascular disorders [1]. This sounds extreme, but all medical professionals ought to agree on a simple fact: we cannot treat coronary disease. We can perform bypass operations, angioplasty, stents, and heart transplants, but these are all palliative emergency measures that only delay morbidity and mortality; they save lives but do not address the problem fundamentally. Undoubtedly, angioplasty and stenting are major innovations in cardiovascular treatment, but restenosis follows. Now, after years of reports on the successful outcome of stenting, we even question whether we should return to medical therapy alone for certain coronary diseases [2].

Is this goal achievable? Could we possibly treat coronary disease as effectively as we learned to treat certain acute diseases – as we treat an acute pneumonia with antibiotics or acute organ rejection with anti-rejection drugs? Why cannot we treat coronary artery disease the same fashion? Prevention via healthy life style works [1,3-5], but it is not what we are investing in. We want to help patients when they become sick. We want to make diseased organs healthy again. So, is coronary disease treatable in general or we are chasing an unattainable dream?

### Subject of analysis

#### Definition of intimal hyperplasia

The subject of my analysis is arterial intimal hyperplasia. This term applies to any cells that form a multi-layer compartment internally to the elastic membrane of the arterial wall and express alpha-smooth-muscle actin, permanently or transitionally [6,7]. The pathology of coronary disease comprises a number of distinct features such as intimal hyperplasia, appearance of foam cells/macrophages and cholesterol buildup, platelet aggregation and thrombogenesis, inflammation etc. These features often overlap and aggravate each other [8], but this analysis focuses exclusively on arterial intimal hyperplasia since it represents a separate pathological entity [9-11]. It is a cell proliferation/differentiation process, representing cellular morphogenesis in its traditional sense [12-14], while cholesterol accumulation and plaque formation is a degenerative process, usually described under the heading "Endogenous substances accumulating in tissues as a result of deranged metabolism" [15]. Although it is worth noting that excessive intimal hyperplasia usually precedes atherosclerosis (appearance of foam cells/macrophages, cholesterol accumulation and plaque formation) [7,10,11,16], analyzing these characteristics together inevitably diminishes significance of correlations [17].

#### Medical significance of coronary artery hyperplasia and history of approach

Arterial intimal hyperplasia (other definitions include arteriosclerosis, neointimal formation, vasculopathy, etc.) contributes significantly to initial (pre-interventional) coronary artery disease [18-20]. We used drug therapy for decades; but since it was not satisfactory, a new state-of-art tool was created – coronary intervention. Nevertheless, intimal hyperplasia appears to be the sole or major devastating pathological remodeling in post-interventional complications after angioplasty, bypass operations or stenting [21-23], and once begun, it is untreatable. We introduced bypass surgery, but intimal hyperplasia keeps growing in the grafted veins and arteries. We introduced angioplasty with balloon dilatation, but intimal hyperplasia grows after vessel stretching. We introduced angioplasty with stenting, but intimal hyperplasia keeps growing through the stents. We introduced stents with the best rational design – radioactive emission – but intimal hyperplasia, together with late thrombosis [24-26], again significantly hampered this innovation [27]. We introduced drug-eluting stents, which retard growth, but intimal hyperplasia continues [28-31]. Intimal hyperplasia threatens literally every known vascular reconstructive procedure and no prophylaxis is available [32,33]. Reports evolved from very optimistic [34] and cautiously optimistic [35] to questioning the long-term effectiveness of coronary intervention [2,36-38].

Common sense tells that tangible factors must cause and perpetuate this devastating hyperplasia pathology. The basis of such an approach is quite obvious. Scientific medicine was founded on fundamental milestones: the discovery of microorganisms and understanding their connection to disease, then the discovery of vaccination/antibiotics followed by successful prevention and treatment of diseases [39]. The historically beneficial model "bacteria → disease → vaccination/antibiotic → cure" was then transformed into "aberrant protein expression → disease → corrected protein expression → cure" model. Owing to the nature of biology, the reduction of problems to simple cause and effect mechanisms is a basic and very effective approach to medical science. Armed with this obvious idea we never stop searching for causes, but the results we have achieved are very far from desirable. Hundreds of thousands of articles and hundreds of monographs have been published, countless scientific meetings held. Every molecule associated with coronary stenosis, soluble or residual, has been thoroughly investigated and characterized and attempts have been made to modulate it, often successfully. The result is the same: we cannot treat the disease. Nevertheless, it is reasonable to suggest that examining factors associated with chronic diseases in "cause – effect" fashion may finally produce a much needed answer, so it should remain the main methodology. Therefore, on the basis of conventional wisdom, we try the same approach again and again.

#### Methodology of research on chronic disorders

There is a valid argument, however, that in chronic disorders we encounter problems that cannot be reduced to simple cause and effect mechanisms [40,41]. Experience shows that the "one protein – one disease" relationship is the exception rather than the medical rule. Usually, chronic disorders result from alterations of normal controls, but the associated altered parameters, although detectable, do not necessarily point to causation or suggest possible approaches to prevention [41-44]. Altered parameters in chronic diseases also depend on numerous factors or variables that are difficult to control and analyze [45]. Nevertheless, the paradigm "one (few) protein – one disease" dominates the scientific study of chronic disorders with organ remodeling. The hope for a "lucky" molecule and "magic bullet", combined with modern state-of-the-art instrumentation, opened the floodgates for competitive data collection. Unfortunately, collection of measurable parameters is widely assumed to constitute knowledge in both medicine and biology, and this is not true [46]. Therefore, we effectively consume our scientific resources by highly competitive data collection, adding to an already overextended collection of disparate factors associated with the disease. New research tools, e.g. studying arteriosclerosis and restenosis in terms of the typical characteristics of transplant immunology, definitely yields new information [47-50], but the theoretical basis for approaches of this kind is not convincing. It actually becomes increasingly difficult to find articles containing particular information, because any given literature search yields thousands of irrelevant references burying a few useful ones. In addition, mixing all associated parameters in any analysis has been shown to diminish the prognostic correlative value of obviously related observations [17].

#### Is coronary arteriosclerosis a treatable condition?

Hypothetically, both "YES" and "NO" are valid answers to the question "are coronary arteriosclerosis and re-stenosis treatable conditions?" The "NO" answer seems more plausible since it receives continual experimental conformation, but we would not wish to choose it for at least three reasons. First, against all odds, we believe that all diseases are cognizable entities and therefore treatable; we also know that some diseases that were completely untreatable in the past came to be understood and cured later. Second, the academic community depends on public funding and the pharmaceutical world is based on profit. The "NO" answer would be collective corporate suicide and is therefore very improbable. Third, all members of our society have a natural desire to remain healthy until death at an advanced age. Therefore, there is a unanimous desire and demand only for the "YES" answer, and we must endorse this no matter how implausible our experience makes it sound. But if "YES" is the only answer, we ought to do something better than before. Otherwise, for how much longer will society be willing to tolerate the ineffectiveness of investment? Not very, according to some scientists.

Some scholars anticipate that research funding for chronic disorders will simply be reduced because of the lack of return and alternative claims for funding [51,52]. This prediction is plausible and extremely worrisome, so why should we not try alternative approaches to the problem?

#### Shortcomings of the traditional approach to coronary intimal hyperplasia

All major hypotheses, and hence approaches to the pathology of intimal hyperplasia, are traditionally founded on the cornerstone question: what causes the pathology? I argue that this question is misplaced because it implies that (a) intimal hyperplasia is a novel pathological phenomenon caused by new mechanisms and (b) the putative cause is not within intimal hyperplasia but external to it. A simple inquiry into arterial morphology shows the opposite is true. The normal multi-layer cellular organization of the *tunica intima *is identical to that of diseased hyperplasia, a standard arterial system design in all placental mammals at least as large as rabbits, including humans [53-68]. Formed initially as a one-layer endothelial lining, this phenotype can either be maintained or differentiate into a normal multi-layer cellular lining, so striking in its resemblance to diseased hyperplasia that we have to name it "benign intimal hyperplasia" [69-71]. However, normal or "benign" intimal hyperplasia, although microscopically identical to pathology, is a controllable phenotype that very seldom compromises the blood supply. It is remarkable that each human heart has coronary arteries in which a single-layer endothelium differentiates early in life to form the multi-layer intimal hyperplasia and then continues to renew itself in a controlled fashion throughout life [61,67,70,72-77]. Although normal intimal hyperplasia becomes bigger with aging [78], very rarely does it grow into a disease compromising the blood supply to the heart. Normal intimal hyperplasia becomes uncontrolled causing impaired coronary blood flow requiring intervention, in only a small fraction of human population [79,80]. Two obvious questions should inform the priorities of our research: (1) what controls are responsible for switching the single cell-layer intimal phenotype to the normal multi-layer intimal hyperplasia? (2) what controls maintain the normal benign intimal hyperplasia?

Differentiation of the *tunica intima *and normal benign intimal hyperplasia are controlled and maintained in vast majority of human hearts. We do not know how this regulation works, but nor do we invest much in its study. On the other hand, in only a small fraction of humans (that could be approximated on the order of 1% [79-81]), this obscure regulation malfunctions jeopardizing life for unknown reasons and we are investing almost all our resources in studying possible causes of such malfunction. Would it not be more logical to approach the problem the other way around? Besides, we already know that even the most rigidly programmed morphogenic processes can deviate under the influence of a whole range of non-specific foreign signals, and it is useless to study non-specific signals to elucidate morphogenesis [82]. Furthermore, judging from the clinical failure of all therapeutic approaches based on elimination of one factor or a handful of factors, it appears that non-specific stimuli are multiple, interchangeable and act in yet unknown combinations. These features make non-specific signals unrealistic therapeutic targets.

#### Origin and consequences of misleading approaches to arterial intimal hyperplasia

All science is about causation. We observe an event and if it is not consistent with our explanatory models, we ask why. In order to ask a question we must see a discrepancy between what is observed and what the model predicts; the observation should be surprising. Is it surprising that the arterial intima expresses and maintains two distinct phenotypes within the same arterial conduit throughout human life, or that one of these phenotypes, normal intimal hyperplasia resembles the disease so strikingly that it has been named "benign intimal hyperplasia" [69-71]? Is it surprising that "benign intimal hyperplasia" is so well controlled that it never turns into disease in the vast majority of humans? In general, not at all!

Medical scientists in mainstream research either do not appreciate these fundamental facts or are simply not aware about them. In consequence, all approaches operate on the assumption that the normal arterial intima is always an "ideal" [83] single-layer endothelium. Even worse, we teach medical students this distorted view. Any standard textbook of histology, e.g. [84-86], along with most monographs on coronary disease, e.g. [87-90], presents arterial morphology this way. The famous "Color Atlas of Cytology, Histology, and Microscopic Anatomy" for medical students by Wolfgang Kuehnel [91], which was translated into all Western languages, does not even include coronary artery morphology, leaving readers with the illusion that it is the same as in any artery of this caliber. At best, some textbooks comment briefly that the intima of elastic arteries may be thicker [92,93], or that the intima of coronary arteries shows the greatest age-related changes [94], still stressing the single-cell layer intimal design. Rare exceptions such as the "Histology for Pathologist"[95], chapter 33 "Blood Vessels" [96] or [97] cannot reverse this general perception because few people read them and do so too late, after this ideology has already been formed.

Common sense leads one to question whether the current disastrous outcome in arteriosclerosis treatment may not arise because the common stock of hypotheses underlying these studies is misleading. These dominant hypotheses are based on two major premises: (1) arterial intimal hyperplasia is a pathology formed *de novo*, due to *de novo *pathological changes in regulation replacing the single-layer intima; and (2) the putative *de novo *causative mechanisms occur outside the site of pathology. This perception is unlikely to change, since we teach students deficient knowledge about arterial morphology and differentiation, making it very likely that the problem will continue to be approached from wrong premises.

#### Some publications allude to intimal hyperplasia under normal conditions but this has little influence on contemporary research

These contentions may be dismissed on the basis of the many articles that discuss normal intimal hyperplasia in regard to arterial pathology, as my opponents argued before, so it is necessary to clarify the point. Some papers do indeed contain allusions to intimal hyperplasia under normal conditions. Some of them make the customary comment that arteries with normal intimal hyperplasia are prone to arteriosclerosis [10,11][67,98,99]. Unfortunately, this research stops short of making any scientific tool from observations. Consider the two most frequently cited. (1) Stary et al., 1992 "A definition of the intima of human arteries and of its atherosclerosis- prone regions. A report from the Committee on Vascular Lesions of the Council on Arteriosclerosis, American Heart Association", published in *Circulation *[10] and in *Arteriosclerosis and Thrombosis *[6], has been cited 365 times. This is a gigantic, detailed study but it lacks even a hint of the notion that studying normal hyperplasia and its regulation can be used as a tool in understanding the disease. (2) Schwartz et al., 1995 "The intima. Soil for atherosclerosis and restenosis" [99], has been cited 586 times. This work actually advocates the opposite idea – that factors/mechanisms causing pathology are new and have nothing to do with the control of normal hyperplasia. Three questions are formulated in the article, underlying the priorities in studying arterial pathology. One of them (#2) addresses exactly the topic of the discussion: "What molecules control neointimal formation?" [99]. This question is asked about pathological intimal hyperplasia or arteriosclerosis. There are no questions in this article about the control of normal hyperplasia or its imbalance. This view is repeated in other publications by the same group, e.g. in the book "Intimal Hyperplasia" [100]. In a section discussing mechanisms and models of restenosis, there is only one line about the similarity between diseased intimal hyperplasia and normal arterial morphology; in contrast, there is plenty of discussion about molecules originating outside the intimal hyperplasia that could control the pathology [98]. Study of normal intimal hyperplasia regulation was not even mentioned in the final section "Future Directions". Therefore, in this matter my opponents appear to confuse two different states of mind: knowing facts as a possession of information; and connecting facts as acquisition of knowledge.

#### Outcomes of the failure to control and prevent arterial intimal hyperplasia: chronic rejection of organ transplants as exemplar

Anyone familiar with the problem knows that failure to control and prevent arterial intimal hyperplasia dramatically affects the outcome of many other disease conditions: peripheral arterial occlusive disorder, graft vascular disease in transplantation, prosthetic vascular failure, etc. A classical example of failed treatment strategy is the management of chronic rejection in organ transplantation. Solid organ transplantation, a relatively new field of medicine, made a tremendous progress in recent decades including surgical techniques, organ procurement, preservation, matching, prevention and treatment of acute rejection, etc. There was one exception: chronic rejection, which still disastrously affects the outcome of transplantation as it did decades ago. In my view, the current failure and lack of feasible solutions to the problem are mainly due to inconsistent and misleading tentative hypotheses underlying the current approaches to graft vascular disease.

A pathology of chronic rejection includes a number of features, but only graft vascular disease forms patterns and is diagnostic [101,102]. In its turn, graft vascular disease may or may not present as venous pathology, arterial inflammatory-necrotic damage, atherosclerotic plaques, or medial or adventitial damage/remodeling. However, it invariably presents as arterial neointimal formation or intimal hyperplasia [101,102], the main manifestation of chronic rejection in solid organ transplantation, less evident in liver [103] and not in lung [101,104,105]. The main causes of graft vascular disease are assumed to be the introduction of alloantigens and an activated immune system [101,106-116]. Although non-immunological factors were considered aggravating and even predictive [116-119], they have never been considered as pathogenetic causes of chronic rejection. Accordingly, our efforts have concentrated on immunological mechanisms for graft vascular disease (GVD).

Because of the prominent and profound arterial pathology in solid organ transplantation, arterial transplant models were introduced to study chronic rejection [120-122]. All these models showed circumferential intimal hyperplasia, similar to the clinical manifestation of GVD, and are widely used to study chronic rejection. As expected, these models were also studied from the standpoint of transplant immunology. Numerous studies based on immunological models of GVD have reported successful abrogation or even prevention of chronic rejection in animal models; nevertheless, this laboratory success has never been translated into clinical progress. As a result, our inability to control chronic rejection, together with an increased shortage of donor organs, has had a catastrophic impact on solid organ transplantation. Because immunological models of GVD still prevail [123,124], it would be helpful to test their logical consistency and fitness to empirical observations.

Since arterial allo-transplantation models are widely accepted for studying chronic rejection, let us consider the well-known fact that identical neointimal formation occurs in human autologous arterial grafting [125-130]. These clinical facts are echoed by experimental observations: everyone who studies animal models of arterial transplantation knows that significant numbers of syngeneic/autologous grafts develop intimal hyperplasia, more often at anastomosis sites, with some groups reporting that 100% of autologous grafts are affected [131]. However, a general consensus disregards syngeneic/autologous anastomosis intimal hyperplasia by examining artery cross-sections from the middle of vascular grafts only. I personally examined more than a thousand grafts in rodent models of arterial transplantation, and also found that anastomosis neointimal formation in syngeneic grafts was very frequent. The pathological patterns of the resultant syngeneic/autologous intimal hyperplasia are identical to those in diseased arterial allografts. Similar to other protocols and in accordance with mine, I evaluated sections from the middle of grafts and disregarded any pathology close to the anastomoses.

These facts lead inevitably to the question: are neointimal remodelings in allogeneic and autologous/syngeneic grafts different in nature or the same phenomenon, i.e. result from the same mechanism(s)? Though at the first glance this question seems redundant, it is very logical. We cannot simply exclude autologous/syngeneic arterial graft pathology from consideration and restrict our analysis to allo-grafting. The only scientific approach to the problem is to incorporate all facts into the analysis and suggest one of these alternatives: either both transformations have distinct mechanisms that coincidentally lead to identical pathology (e.g. structural convergence), or intimal hyperplasia in allo- and autologous/syngeneic grafts result from the same mechanism. Conventional wisdom tells that we have to select the simplest explanation [132]. Therefore, unless otherwise proven, we have to suggest that the same cause underlies intimal remodeling in both autologous/syngeneic and allografts, just for the sake of logic. Because no alloantigens are involved in autologous/syngeneic arterial transplantations, it is logical to ask a further question: why did we assume in a first place that introduction of alloantigens and activation of the immune system causes intimal hyperplasia in transplanted arterial allografts, i.e. GVD, i.e. chronic rejection in solid organ transplantation?

The answer is obvious: because GVD occurs after allogeneic organ transplantation and the same introduction of alloantigens causes a profound phenomenon known as acute rejection. Indeed, allografts undergo acute rejection, and to explain this, an idea (due to Sir Peter Medawar and Sir Frank Burnet) about how the immune system rejects or accepts tissue transplants was applied. On the basis of this concept, braking through anti-rejection drug therapy was created (for review see [133,134]). Nevertheless, although historically obvious for transplantation, the allo-immune hypothesis of chronic rejection has produced no progress, i.e. experimental testing has failed. All approaches to treatment based on the allo-immune hypothesis of GVD failed to generate progress, and unlike acute rejection, the rates of chronic rejection have remained largely unchanged over the decades [135,136]. As a result, our inability to control chronic rejection, together with an increased shortage of donor organs, has had a catastrophic impact on solid organ transplantation, yet we are still using the same approaches to the problem.

In short, the alloimmune hypotheses of GVD have failed experimental tests, have a logical flaw and do not fit observations. Rationally, alloimmune models should be rejected. It does not matter that we do not know yet the cause of uncontrolled intimal hyperplasia in the complication named "chronic rejection". We simply have to refute the failed hypothesis, suggest others and test them. Freeing analysis from pathogenetic bias is not just logical, it is imperative for scientific progress. A hypothesis is a tentative assumption made in order to draw out and test its logical or empirical consequences [137]. Therefore, asking questions from the standpoint of inconsistent and failed hypothesis can only generate misleading answers. Nevertheless, the failed hypothesis still prevails [123,124].

#### Main-stream research on arterial intimal hyperplasia continues to base approaches on inadequate hypotheses

I included the foregoing synopsis of chronic rejection for two reasons. First, I have studied chronic rejection over the last 15 years with a growing realization that there is logical inconsistency in this field, and this was a topic of the first version of this analysis. Secondly, I see it as a very clear example of the disconnection between observations and scientific reasoning on the one hand and explanatory hypotheses on the other. Therefore, it is not just a failure of certain treatment strategies, it is much worse – it is a persistent failure to address the problem. Everything that could be considered as part of immune regulation or remotely associated with it has been suggested as cause and thoroughly tested, and it has failed to produce results. This claim does not even require references; it covers everything from large domains such as innate and adaptive immunity, cellular and antibody-mediated immune responses, to smaller domains such as soluble and membrane-associated antigens, complements, etc. Whatever has been suggested as causation within immunological models has failed experimental tests. Did we abandon this hypothesis? Not at all, it is still the main approach to chronic rejection, though it is now customary to speak of a "cytokine milieu". We now seem to be working with hypotheses that are not falsifiable.

#### To date, arteriosclerosis research has taken no cognizance of fundamental facts about arterial morphology, and these facts must be re-discovered

While working on this analysis I came across one recent publication with mixed feelings. A research group from Boston published an extremely important report that is worth quoting. For the first time in modern periodical publications, clinical researchers put a much needed question mark in the title of this article: "Cardiac Allograft Vasculopathy: Real or a Normal Morphologic Variant?". Houser and co-authors [138] wrote:

"Naive coronary vessels may appear to have intimal thickening histologically characteristic of cardiac allograft vasculopathy (CAV)." *from abstract-VS.*

"However, as illustrated in Figures 1 and 4, in a notable number of vessels in naive and native hearts, the smooth muscle cells' expanding intimae lacked this neatly regular pattern. Ignoring this finding could result in a diagnosis of CAV when, in fact, no CAV is present."*from discussion-VS.*

Considering that most researchers in cardiology still believe that normal intimal hyperplasia is confined to closure of the *ductus arteriosus *[139], the significance of this report [138] for the entire field of arterial pathology cannot be overestimated. On the other hand, it clearly indicates that the most advanced research groups in the field are not fully aware of the normal coronary artery phenotype [53-55,57-68,70,72-74,76-78,140-149] or of the possible implications of this normal regulation for pathology.

One might suppose that, since the publication of this breakthrough report [138], we should expect changes in the perception of the disease and approaches to its solution. However, I remain skeptical. I wish to be wrong, but judging from history, it is very unlikely.

#### Concern has been expressed about the lack of attention to fundamental properties of arterial structures in medical studies

Two decades earlier, the renowned UK pathologist Collin L. Berry wrote in chapter 3 ("Organogenesis of the Arterial Wall") of the monograph "Diseases of the Arterial Wall" [150], original French edition "Maladies de la paroi arterielle" [150], in the synopsis "Exceptional areas in vascular development":

"There is a considerable body of literature on the significance of what have usually been described as "endothelial cushions", mainly in coronary arteries (see Robertson (44) for review of early literature). Robertson concluded that the lesions, which could be found in other arteries, were not related to subsequent atherosclerosis but were normal growth phenomenon. These studies however, and the subsequent careful work of the Velicans (55,56), have been ignored in recent years." [151]

Experience shows that that Berry's assessment was not only correct, but unfortunately predictive of the following twenty years. But are we experiencing *déjà vu*?

More than five decades previously, Nikolay N. Anitschkow wrote in the chapter "Experimental arteriosclerosis in animals" of the book "Arteriosclerosis. A Survey of the Problem", edited by Edmund V. Cowdry [152], in the section subtitled "Interpretation of experimental intimal thickening":

"... in evaluating the significance of the thickening of the intima, as observed by various authors, it is important to remember that thickening of the intima also occurs in experimental animals *as a purely physiological phenomenon in the process of aging*. In this respect, the arteries of some animals exhibit almost the same conditions that are observed in human arteries, as may be seen from Miss Wolkoff's investigation (1924). In the view of the fact that some authors mentioned above did not pay any attention to this circumstance, the experimental results reported by them can be accepted only with very great reservations" (pp. 275–276).

Further, in subchapter IV, "Spontaneous arterial changes in animals", Anitschkow wrote:

"Another circumstance that should not be left out of account by any author interested in the experimental induction of atherosclerosis is the frequent occurrence of *spontaneous arterial changes *in certain species of animals as described in chapter 6". (p. 276) [153].

Let us not forget that N. Anitschkow (alternatively spelt "Anichkov") is a Russian pathologist famous for his seminal theory on the "cholesterol pathogenesis" of arteriosclerosis, and his pioneering work on arteriosclerosis modeling [153-155]. Anichkov's work is considered among the greatest medical discoveries of the 20th century [156,157]. Can we find any consequence of these straightforward notions written by one the most influential scientists in the field? See above.

#### The pioneering work of Richard Thoma on normal arterial intimal hyperplasia

But if we wish to trace the origin of this conceptual approach to arterial design, we have to look back more than a century to the work of Richard Thoma of Heidelberg, a founder of the modern arterial pathology. Over more than forty years, Thoma published observations and hypotheses in series of articles in leading pathology journals about the resemblance between normal intimal hyperplasia and arteriosclerosis in the umbilical artery, ductus arteriosus, different segments of aorta, coronary artery and other arteries. Thoma hypothesized that arterial intimal thickening is a physiological adaptation to changing haemodynamic demands [53,54,140]. In his publications Thoma uses the German "Neubildung" or "Gewebsneubildung" to describe new (tissue) formation without transformation, i.e. normal hyperplasia. To describe diseased hyperplasia, he adds "Angiosklerose" and "Angiomalacie".

In "Über die Intima der Arterien", Virchows Archiv, 1921, 320,1:1–45, among many descriptions of normal arterial intimal hyperplasia at various sites, Thoma writes in the Conclusion (pp:44–45):

"According to these general effects, the neoplasia of connective tissue which occurs following birth in the umbilical bloodstream, appears as a necessary consequence of the conditions present. The closing of umbilical arteries and the Botallian duct produces a considerable increase in the amount of blood flowing through the descending aorta and Art. iliacae comm. per unit of time, since the peripheral areas of circulation of the lower extremities and the rest of the body at first receive no greater amounts of blood than before....".

"....The retardation of the stream thus triggers, according to the first histomechanical laws of bloodstream, a neoplasia in the intima, which narrows the opening of the vessel. Through this increase in thickness of the intima, on the one hand, and on the other hand, as a result of growth of the media in these arteries, delayed by the tonic narrowing, normal speed of peripheral bloodstream is restored during the period of 2 to 5 years....

"The exact same relationships arise in angioslerosis, with the difference that vessel tonus is destroyed as a result of angiomalacia. Angiomalacia becomes the cause of diffuse and circumscribed, passive distension of vessel walls through the pressure of blood. These distensions of the arterial wall result in greater or smaller retardations of peripheral bloodstream, which, under the exhausted tonus of the media, lead to diffuse and circumscribed neoplasia in the intima. This neoplasia in the intima is in the beginning at times rich in elastic and muscular elements, when through blood pressure or as a result of widening of the opening the tension of the wall is increased. When, however, mechanical tensions of the vessel wall are moderated, either through hypertrophic thickening of the media or though a strong increase in the thickness of the intima, then the endothelium goes on to produce primarily connective structures, which correspond to moderated mechanical tensions." [54] (Translation from the German by [158].

#### Confirmation of Thoma's hypothesis of the remodeling of normal intimal hyperplasia

More recently, Thoma's hypothesis of the remodeling of normal intimal hyperplasia has been further investigated and subjected to experimental testing. It has been unequivocally confirmed in a number of elegant studies [159-173], leading to advanced modeling such as the Glagov and Kamiya-Togawa models. A very powerful conformation of the "slow flow" effects on expansion of hyperplasia and arterial narrowing was reported by Karino-Goldsmith group [174-181]. Results of this group, obtained in fascinating experiments on transparent arteries with preserved geometry, including human arteries, directly showed that disturbed or slow flows are associated with excessive hyperplasia [174-181]. Significance of precise direct observations on fluid mechanical factors influencing intimal hyperplasia, and thereby connecting the models to coronary diseased hyperplasia, cannot be overstated. This seems a particularly striking example of the disconnection among scientific fields that are in effect concerned with the same phenomenon; indeed, notable scientists have contributed to this work [164,169,171,181,182] and published it in journals that are dedicated to arteriosclerosis.

#### The past 50–60 years have yielded no new conceptual ideas about arterial intimal hyperplasia pathology and we no longer expect any

Richard Thoma was the first to enunciate a conceptually motivated approach to the problem. In my view, this was the foundation of his tremendous personal achievement in the field of arterial pathology, and for the extremely important observations and conclusions made by scientists whom his ideas inspired [159-161,163-173,181-184,187,188].

Even during his lifetime, Thoma had been criticized for omitting lipid depositions in intimal hyperplasia from his model [189]. Indeed, lipid deposition in intimal hyperplasia had already been noted by Rudolf Virchow [190] (cited from [189]). This phenomenon inspired Anitschkow's work [153-155], opening a new chapter in the study and prevention of arterial disease. Again, in my view, a conceptually motivated approach to the problem was the driving force behind Anitschkow's achievement and the tremendous clinical success that came from it.

However, scientific reality comprises a natural sequence of events; and – as has happened before – any productive theory may cease to be useful when applied beyond its limits. Even worse, it may become a dogma monopolizing research and slowing progress [40,41,191]. The "cholesterol" hypothesis still is the best explanatory model for certain clinical observations, but not for all. It took a long time before a prestigious medical journal – the NEJM – became open to discussion about the "cholesterol monopoly" [192-194]; though surprisingly, previous publications challenging the "cholesterol" dogma [195-197] were not mentioned. My point, however, is that Anitschkow's work was, and inspired, a conceptually motivated approach to the problem, and that is why it resulted in tremendous success.

No new conceptual ideas seem to have arisen during the past 50–60 years of study of arterial neointimal formation in either field of medicine. More dangerously, we have grown accustomed to having no new ideas. I share the opinion that the idea is more important than the experiment [198], and without drastic changes in the perception of the problem, progress is very unlikely. I proposed a hypothesis aimed at incorporating all facts related to intimal hyperplasia, and analyzed the problem from the viewpoint of established biological knowledge.

### A unifying hypothesis

#### Observations on intimal hyperplasia that may be connected and explained by the hypothesis

First, I shall enumerate all the facts that I suggest are interrelated and should therefore be explained by one hypothesis.

Arterial intimal hyperplasia (IH) is a distinct arterial tissue formation or arterial phenotype that manifests as follows:

(1) IH appears in the inner compartment of the arterial wall, the "intima", as a multi-cellular layer as distinct from the single-cell-layer endothelial lining.

(2) IH always occurs under normal conditions in all air-breathing vertebrates from lungfish to mammals in one strictly predetermined arterial location: the sixth pharyngeal arch artery or its derivatives (the *ductus arteriosus*, otherwise known as the Botallian or Botalli duct). Closure of the *ductus arteriosus *separates the pulmonary and systemic arterial blood flows, permanently or temporarily [199,200].

(3) IH always occurs under normal conditions in the uterine arteries in placentals of various taxonomic orders [201-212], and probably in all placentals. It participates under normal conditions in the closure of umbilical arteries in humans [213-215]. This closure has been studied in the distal part of the umbilical cord, and I suggest that it is the main mechanism sealing the vessels in the proximal part, i.e. the navel.

(4) IH always occurs as the standard design of major arteries in all placental mammals at least as large as rabbits, including humans [53][54-67,72][73-76,78,138][140-149][150-152][153]This morphogenesis does not have a completely predetermined location, but occurs most frequently in arterial sites proximal to highest blood pressure [64]. This arterial phenotype possesses great dimensional variability in respect of location, vascular length affected and intimal width.

(5) IH normally occurs and increases with age in at least two peripheral limb arteries in humans [216] and probably in other big arteries [78].

(6) IH occurs under normal conditions as the standard arterial system design in two other taxa of vertebrates: birds and marsupials [56,64,217,218].

(7) IH also occurs under normal conditions as the standard design of low limb veins in humans [219].

(8) Under disease conditions (both clinical and experimental), IH is manifest in vessels of all types, including prosthetic, if they constitute part of the arterial system. These manifestations show striking variations in location and extent, and the associated disease conditions show similarly striking variations in nature and magnitude. These pathological hyperplasia formations occur as:

(1) spontaneous excessive intimal formation at normal arterial hyperplasia sites (e.g. coronary artery), carotid artery [220] and aorta, more often close to the *ductus arteriosus *[221-223];

(2) spontaneous neointimal hyperplasia formations at sites that normally express the single-layer intimal arterial phenotype (e.g. peripheral arterial disease, more often in limbs [224-228], mesenteric artery system [229,230], or sometimes in multi-organ arteries [231] together with aortic coarctation [232]);

(3) neointimal hyperplasia formation of autologous arterial grafts;

(4) neointimal hyperplasia formation of autologous venous grafts in arterial location;

(5) neointimal hyperplasia formation occurring in response to local insults to arteries *in situ*, regardless of the original intimal phenotype. The nature and magnitude of the insults are extremely variable;

(6) arterial neointimal hyperplasia formation resulting from any solid organ allo-transplantation, except lung;

(7) neointimal hyperplasia formation on the inner surface of prosthetic vascular grafts, bare [233,234] or pre-seeded with endothelial cells [235-238];

(8) arterial neointimal hyperplasia formation after cessation of blood flow [239].

#### Hypotheses about arteriosclerosis and restenosis that fail to incorporate normal intimal hyperplasia and consider only the pathology are logically inconsistent

In my view, this logical flaw generates misplaced questions and accumulates misleading answers. For this reason I omit discussion of other traditional hypotheses of IH, e.g. the inflammatory hypothesis of arteriosclerosis and restenosis [240-247], since there is no inflammation behind normal intimal hyperplasia. The alternative assumption – that an undetectable degree of subtle inflammation always exists in arteries – ultimately makes such hypothesis unfalsifiable and thereby useless.

#### Origin of cells forming arterial intimal hyperplasia

The origin of cells forming arterial intimal hyperplasia have been shown to be:

(1) residual endothelial cells;

(2) residual smooth-muscle cells;

(3) residual adventitial cells [248];

(4) residual transdifferentiated cells [249];

(5) different progenitor cells, residual or bone-marrow, including neural-crest-derived progenitors [250];

(6) cells of either donor or recipient origin or both in transplantation models;

(7) cells of unspecified origin except residual smooth-muscle cells [251-253], based on the fact that in these models, all residual smooth-muscle cells die before hyperplasia formation begins.

These facts about intimal hyperplasia (different normal and pathological manifestations, as well as different cell origins) can, in my opinion, have only one explanation.

#### Hypothesis about arterial intimal hyperplasia

The hypothesis states that:

Arterial intimal hyperplasia is a phenotype or biological trait that has evolved and been selected as normal arterial morphogenesis, initially as an adaptation facilitating air breathing in anamniotes (forebears of lung fish), then, as the two circulations separated and the lung was bypassed during amniotic embryogenesis, facilitating closure of the *ductus arteriosus *after hatching in amniotes (forebears of reptiles), and then as an adaptation to increasing arterial blood pressure (increased body weight, variations in anatomical design, upright body posture, etc.), to preserve arterial integrity and to regulate blood flow to comply with local physiological demands. The cellular source for this morphogenesis may be any cells that colonized and survived in the intimal compartment. These comments are not new; they are stated here to ensure the logical coherence of what follows.

Since individual variability is a fundamental property of all species, this morphogenetic reaction cannot be absolutely pre-programmed in terms of either location or extent, except for a few locations – the *ductus arteriosus *and the umbilical and, probably, uterine arteries.

In both phylogeny and ontogeny, vertebrates display great variation within and between taxa, affecting individual body weight (exceptions are [254,255]), posture, anatomy, behavior pattern, etc.. These variations are ultimately associated with variations in arterial pressure, even within homogeneous groups of the same species [256-259]. We know that significant variations in blood pressure correlate with variations in normal intimal phenotype; specifically, high blood pressure correlates with normal intimal hyperplasia in arteries proximal to heart [53-56,58-67,72-78,138,140,153,160,260][261-264][265]. What mechanisms could control this morphogenesis? Obviously, the requisite information cannot be controlled by cellular DNA alone, for two reasons: (1) this morphogenesis occurs in response to positional forces in the arterial system, which cannot be strictly predetermined for any given organism; and (2) it is facilitated by cells with different differentiation potentials. The only logical solution is that in addition to genomic information, (1) the arterial system itself instructs the intimal phenotype, (2) this information must be arranged in certain patterns along the heart-periphery axis, and (3) under normal conditions, local expression of a specific phenotype depends on the hydrodynamic properties of the blood flowing in contact with the intima.

This mechanism was initially proposed by Thoma [53,54,140] and more recently tested, confirmed and further elaborated [159-161,181,266,269]. Together, these facts offer a sound explanation of how the intimal hyperplasia phenotype arises proximal to the heart in the normal arterial tree, depending on the hemodynamics of blood flow, and changes with location along the heart-periphery axis. However, neither the Thoma's original [53,54,140] nor adapted [176,180-184] models can explain pathological hyperplasia, clinical or experimental, that is not preceded by changes in hemodynamics and shear stress, nor can they explain intimal hyperplasia in prosthetic vessels. However, the Thoma, Glagov and Kamiya-Togawa models and Karino-Goldsmith' observations offer a consistent explanation for pre-interventional *in situ *diseased hyperplasia (arteriosclerosis) as well as for the beneficial effects of cardio-vascular exercise [270,271].

#### Disease-related arterial intimal hyperplasia not preceded by changes in hemodynamics and shear stress

To explain disease-related hyperplasia that is not preceded by changes in hemodynamics and shear stress, I hypothesize that the arterial blood-tissue interface itself (as a topological entity) imposes properties that support the development of intimal phenotypes, initiating mechanisms of cell selection and intimal morphogenesis. This morphogenesis could be directed to the formation of either a single-cell-layer intima ("ideal intima") or multi-layer cellular compartment (intimal hyperplasia). We already know that cells of different origin can form intimal hyperplasia. The same is true for single-cell-layer intima. The hypothesis suggests that any cells capable of colonizing the arterial blood-tissue interface, naturally or in remodeling, acquire by default the capacity to activate genes that are necessary for producing intimal phenotypes. Note that "arterial blood-tissue interface" is defined differently from the traditional "blood-tissue interface", i.e. endothelium [272]. In my model, the term denotes the topological area where blood flow meets surrounding structures, and it includes descriptions such as "basement membrane on which the inner cell lining of vessels rests" or "proteins, glycoproteins and other molecules, including artificial ones, that appeared in fixed positions and form structures in contact with the moving blood. This includes dead vessel wall, prosthetic vascular grafts, autologous and allogeneic vascular grafts, and naïve arterial vessels in any location.

The assumption that the arterial blood-tissue interface facilitates the formation of intimal phenotypes arises from the endothelialization and hyperplasia formation in vascular prostheses, and from observations on intimal formation after initial necrosis of an entire arterial wall in animal models [251-253]. We also know from nascent vessel formation that angiogenesis and blood formation are reciprocally-inducing events [273-277]. Some observations also indicate vessel-related positional information, in traditional cell biology models and in pathology [200,262,278-281]. Recent *in vitro *experiments [186-188] suggested that blood flow (a moving fluid) possesses sufficient information to invoke specific endothelial differentiation and vascular development. The particular properties of blood flow that initiate vascular differentiation remain unidentified, but the phenomenon of blood flow-dependent vascular morphogenesis is clearly apparent [185].

#### Any cell selected by its ability to colonize the arterial blood-tissue interface acquires the ability to express either intimal phenotype, regardless of cell origin

Therefore, I suggest that any cells that are capable of colonizing the arterial blood-tissue interface (i.e. attaching and surviving) acquire the ability to form an intimal phenotype. This acquisition of ability entails the activation of gene regulatory cascades for expressing intimal phenotypes. Under this hypothesis it does not matter whether the cells are of donor or recipient origin, resident or blood-borne or trans-differentiated, etc.; nor does it matter whether the vessel is a residual artery, natural or prosthetic graft. Once selected by their ability to remain at the interface, or prompted to differentiate to such state from residual sources, cells acquire the ability to enter intimal morphogenesis. The cells that appear in the innermost position are endothelial cells, by topological default and regardless of heterogeneity [282]. After cells have colonized the arterial blood-tissue interface, basal membrane components, regulatory molecules in the blood and the mechanical properties of blood flow can further direct intimal differentiation [186-188,283,284].

#### The disease state involves no new mechanisms that stimulate the millions of cells constituting the intimal compartment simultaneously, but pre-existing normal mechanisms could be unbalanced by various non-specific or triggering stimuli

Furthermore, the hypothesis suggests that the basic phenotype, i.e. the single-layer endothelial lining, is dominant because the mechanism for producing a multi-layer phenotype is normally suppressed. Thus, there is a balance between positive and negative processes, a dual control, which is common in biology. Under normal conditions, the mechanism for producing a multi-layer phenotype or intimal hyperplasia could be activated by mechanical forces or by the positional information associated with such forces. But in normal conditions, even activated multi-layer morphogenesis is controllable, presumably under negative regulation. For disease conditions, malfunction of either regulatory arm would ultimately result in imbalance, which, as we know, is always manifesting in uncontrolled intimal proliferation. Thus, the hypothesis implies that the disease state involves no new mechanisms that stimulate the millions of cells constituting the intimal compartment simultaneously, but pre-existing normal mechanisms could be unbalanced by various non-specific or triggering stimuli.

#### Relationships between arterial hyperplasia remodelings of different origins

It may be objected that unrelated facts have been conflated, and that relationships that do not exist in reality should not be investigated; in other words, that my quest rests on a false premise. Certainly, it may be argued that normal coronary hyperplasia has nothing in common with hyperplasia in prosthetic vessels, and GVD is unrelated to peripheral arterial occlusive disorder, or to the *ductus arteriosus*, etc. It is a valid argument, but before responding I would like to ask more questions that we usually avoid:

Is intimal hyperplasia after coronary interventions (restenosis) the same phenomenon as pre-interventional coronary intimal hyperplasia?

Is pre-interventional coronary intimal hyperplasia is the same phenomenon as intimal hyperplasia in peripheral arterial occlusive disorder?

Is intimal hyperplasia after coronary angioplasty the same phenomenon as cardiac graft arteriosclerosis?

Is normal coronary hyperplasia the same phenomenon as post-transplanted cardiac arteriosclerosis?

Is intimal hyperplasia in a transplanted heart is the same phenomenon that affects a transplanted kidney?

Is intimal hyperplasia in autologous vascular grafts is the same phenomenon that affects allogeneic vascular grafts?

Is intimal hyperplasia of the *ductus arteriosus *the same phenomenon as intimal hyperplasia in autologous/allogeneic vascular grafts?

Is intimal hyperplasia in autologous/allogeneic vascular grafts the same phenomenon that causes failure of synthetic vascular grafts?

Do we believe that the diseased intimal hyperplasia we study in experimental models is the same phenomenon we deal with in the clinic?

Do we believe that intimal hyperplasias in different experimental models represent the same phenomenon?

Do we believe that the normal hyperplasias that occurs in the arterial systems of three taxa of vertebrates (birds, marsupials and placentals) are the same phenomenon?

Are all the above morphogeneses the same phenomenon or are we dealing with morphological convergence?

Although these questions may seem redundant or paradoxical, they have to be answered because they constitute a quest for similarity, analogy and homology. This is the most important quest in biology, for the following reasons.

#### Determination of analogy and homology in biological science

Medicine is a part of biology. When the nature of a biological characteristic (trait or pathological pattern) is obscure or unclear, the main approach is to search for possible correlations between multiple observations, including an event of interest. When correlations are found, the next step is to examine whether they are analogous or homologous in nature.

Analogous traits have common function but originate in very distant phylogenetic taxa/structures. The wings of insects and the wings of birds are analogous traits and different genomic mechanisms are responsible for their development and differentiation. In contrast, homologous structures evolved from the same primordial structure in a common forebear. For example, the wings of bats, the pectoral fins of dolphins and the arms of humans are homologous owing to their shared ancestry. The same gene expression cascades can be tracked in the development of homologous structures, while non-homologous (analogous) structures, although similar in function, are result from different mechanisms. Consider the following: one and a half century ago, Alexandr Kovalevsky, a Russian founder of comparative embryology, demonstrated similarities in embryonic development between *Amphioxus lanceolatus*, tunicates and vertebrates [285]. Kovalevsky considered these similarities homologous and unified all three taxa in one phylum, Chordates, placing Amphioxus in the ancestral position. In 1994 a comparative analysis of Hox gene clustering in mammals and amphioxus revealed similar genomic organization, showing that indeed amphioxus is a living ancestor of mammals [286]. Classifying a set of observations as homologous or analogous is the most valuable tool in biology. The entire Darwinian concept, as well as modern biology, is based on determinations of homology.

#### The intimal hyperplasia phenotype is a selected biological trait and should be approached in terms of homology

A biological characteristic that occurs in the same anatomical conduit (arterial intima) of almost all related species, and is manifest in pathological states in these species, cannot be an accident and is very unlikely to result from unrelated mechanisms. All biological understanding tells that it is a selected trait. The alternative assumption simply denies the past 150 years of biological science together with Darwin's concept. In my view, the pronouncement that "Nothing in Biology Makes Sense Except in the Light of Evolution" [287] constitutes a valuable scientific tool and not just a political statement.

Therefore, my hypothesis rests on fundamentals of biology. It also rests on the knowledge that if a unifying explanation for a set of presumably related observations exists, that explanation should be given priority for experimental testing rather than a number of separate explanations, one for each observation. The application of intuitive knowledge such as the Principle of Parsimony has certain limitations in science, including that in traditional biological modeling [288], but it is still a useful guide in formulating hypotheses.

#### The analysis has certain implications and offers scientific tools for the study of the disease

What scientific tools does my analysis provide, and what implications does it offer? The most important implication is: we have to change all our approaches to the problem drastically, since they omit fundamental biological evidence and have been shown to be fruitless and misleading. This topic has been considered in great detail by philosophers of science [39-41,191,289] and need not be analyzed here, but we ought to stop deceiving ourselves. We ought to stop pretending that measurable associated parameters collected from diseased patients constitute causal information about diseased intimal hyperplasia. Our experience and accepted hypotheses have not produced feasible candidates for such causation. Knowing where to go and where not to go is essential. We have been testing the same failed hypotheses and using the same fruitless approaches recurrently for decades. Billions of dollars have been spent in the attempt to understand what could possibly unbalance an obscure mechanism, and not even a tiny fraction of this funding has been spent on studying the mechanism itself. We ought to stop the endless arguments about the origin of cells in diseased hyperplasia, because this quest is pointless. We ought to admit that statements typically found in articles and grant proposals such as "causative mechanisms of transplant vasculopathy are not completely understood" can only deceive. We do not have a hint about the causative mechanisms. Therefore, titles such as "fighting coronary disease", "fighting restenosis", or "fighting chronic rejection" sound very attractive to the public and for fundraising, but they are misleading from both medical and scientific standpoints. We ought to use biological knowledge and approaches to study diseased hyperplasia because we are dealing with a biological trait.

In fact, these various observations can no longer be viewed as coincidental, yet no one seems willing to verbalize the problem. As Wallitt *et al. *put it: "Therefore treatments meant to bypass vessels are themselves affected by the very malady they are deployed to treat; it would seem that biology is not without a sense of irony" [290]. I perceive the problem quite differently: when normal intimal hyperplasia is considered as a biological trait and normal IH in coronary arteries as a basic phenotype, and in the light of pre-existing IH in the vascular grafts we use for bypass [219], the real and very sad irony stems not from biology but from our inability to notice and understand all these messages that biology sends to us.

#### We must also urgently revise the way we teach arterial histology and pathology to medical students

This revision is not about educational methodology, it is simply about teaching facts and the omission of facts. If leading medical scientists are harboring such dramatic misconceptions about normal coronary artery design and are re-discovering obvious facts, as evident from [138], then medical education itself is jeopardizing progress. I feel it necessary to re-emphasize my point here: I think that Houser and co-authors [138] have made the most important observation ever in the field of transplant arteriosclerosis, but this later admiration does not negate the former evaluation. The view that diseased coronary hyperplasia is qualitatively different from the normal coronary hyperplasia is no longer tenable.

#### Predictions of the hypothesis and priorities in intimal hyperplasia research

My hypothesis predicts that the gene regulatory cascades governing this morphogenesis in its different manifestations, including diseases, are homologous. If identified, a real regulatory cascade rather than numerous transposable non-specific signals would be a therapeutic target. It also predicts that coronary arteries in animals of more than a certain size should form a multi-layer intimal compartment regardless of taxonomic position. For example, a capybara, the biggest living rodent reaching body weights up to 70 kg [291], should have normal intimal hyperplasia in its coronary arteries. This hypothesis, in addition to logic, internal integrity, appeal to common sense and support from different bodies of knowledge, possesses one essential feature: it is falsifiable.

In the Background section of this article, I stated that two questions should inform the priorities of our research: (1) what controls switch the single cell-layer intimal phenotype into normal hyperplasia? (2) how is normal (benign) hyperplasia maintained?

Do I know the answers to these questions? Unfortunately, the answers to these questions remain unknown; I can only offer a hypothesis. But I am convinced that as long as we approach the problem from false premises, we are fated to accumulate misleading and fruitless answers.

It is very unproductive to approach the problem from the point of view of information we do not know yet, or what other molecules could be studied. From what we have learned about proteins that show deviant expression associated with the disease, of which there are already dozens, it is literally impossible to test even a fraction of the possible combinations. I think we can use our existing knowledge about the subject to forge useful tools. I strongly believe that, collectively, we already know enough to approach the problem from different and more productive viewpoints if we could overcome the information gap between medicine and biology, and between the different fields of medicine that are in effect studying the same phenomenon. We have to put together all the facts and suggestions currently stored in different knowledge clusters. I hope that my analysis may initiate scientific exchange between different fields and facilitate approaches to the problem, although its complexity and magnitude requires nothing less than a community-wide effort. We cannot afford to allow inertia to overtake our research. Diseased intimal hyperplasia, the main pathological manifestation in a variety of arterial disorders, continues to be the world's leading cause of death.

## List of abbreviations

GVD – graft vascular disease

IH – intimal hyperplasia

## Competing interests

The author(s) declare that they have no competing interests.
